# Supplementary material for: The spontaneous neoantigen-specific CD4+ T cell response to a growing tumor is functionally and phenotypically diverse
Source: bioRxiv. 2025 Mar 29:2025.03.25.645281. Preprint. [Version 1] doi: 10.1101/2025.03.25.645281 (PMC11974889; doi:10.1101/2025.03.25.645281)
Supplement: Supplement 1 — Supplemental figure 1: Therapy decreases the frequency of NeoAg-specific FoxP3+ cells a. Representative FACS plots of tumor-resident tetramer-positive CD4+ T cells from untreated and vaccinated animals taken 18 days after tumor inoculation. Supplemental Figure 2: Different activation states for total and CLTCH129>Q-specific cells. a. UMAP with grouping based on activation states determined by Seurat-based clustering originally pictured in figure 4b. Group 0 is primarily composed of tetramer positive cells while groups 1 and 2 are composed of tetramer negative cells. b. Relative CD44 expression of the three groups seen in panel a. c. The frequency of each subset identified 18 days after tumor inoculation through flow cytometry in panel 2c and 17 days after tumor inoculation through single cell genomics in figure 4c Supplemental Figure 3: CD90.1 vs TCR expression a. Flow plots comparing CD90.1 to the TCRVß gene contained in the TCR1 construct in CD4+ T cells that underwent either mock transduction (left) or that were transduced with the construct containing TCR1 (right). Supplemental Figure 4: Transduction efficiency of adoptively transferred cells. a. CD90.1 expression versus tetramer binding for the four sets of adoptively transferred cells used in figures 6f and 6g analyzed before injection Table 1: List of the TCRs Table 2: Expression levels and tetramer binding by TCRs [file media-1.pdf]

Supplementary Figure 1

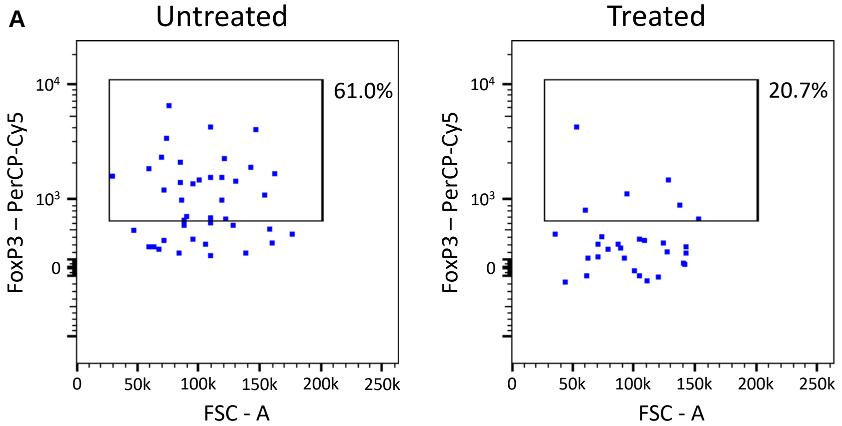

Supplementary Figure 2

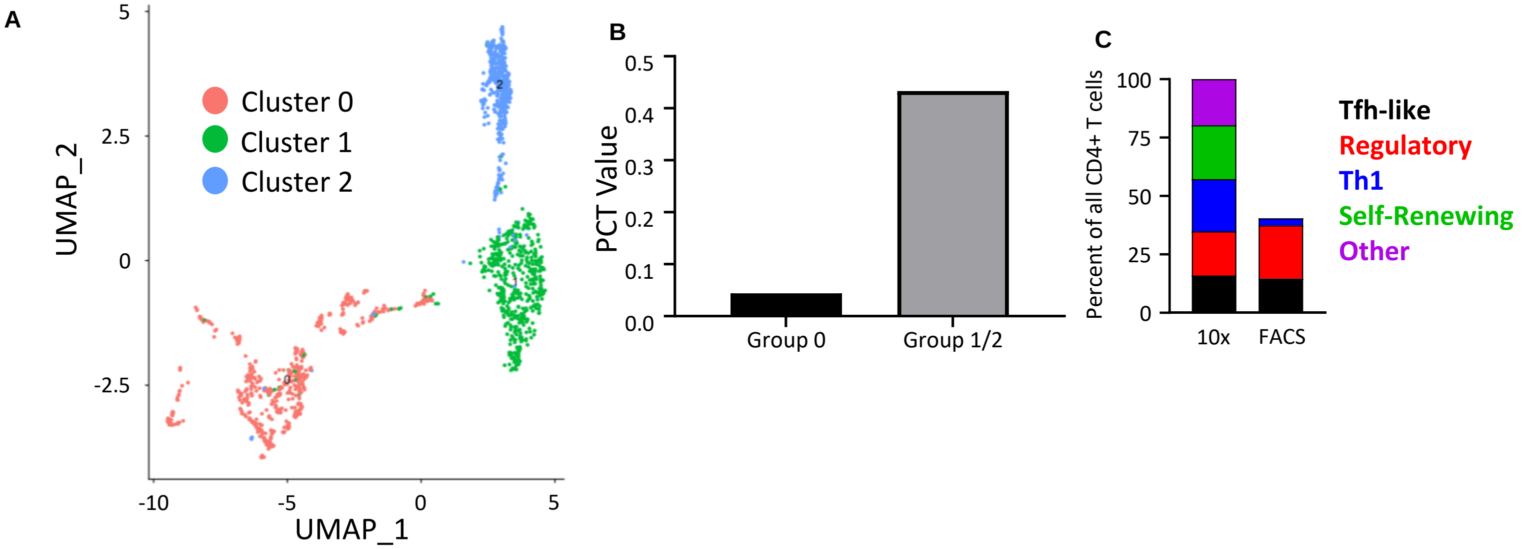

Supplementary Figure 3

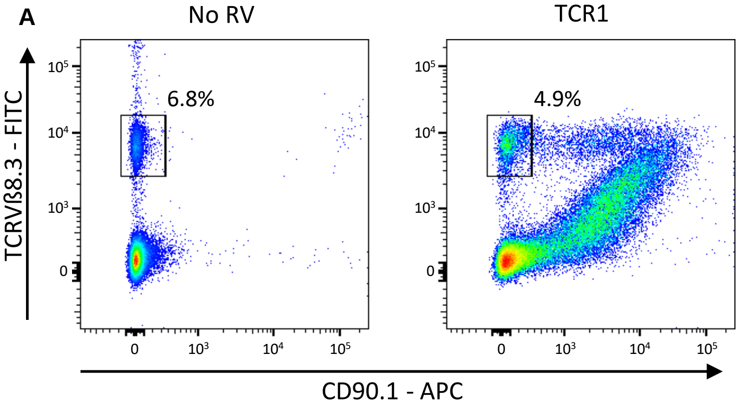

Supplementary Figure 4

A

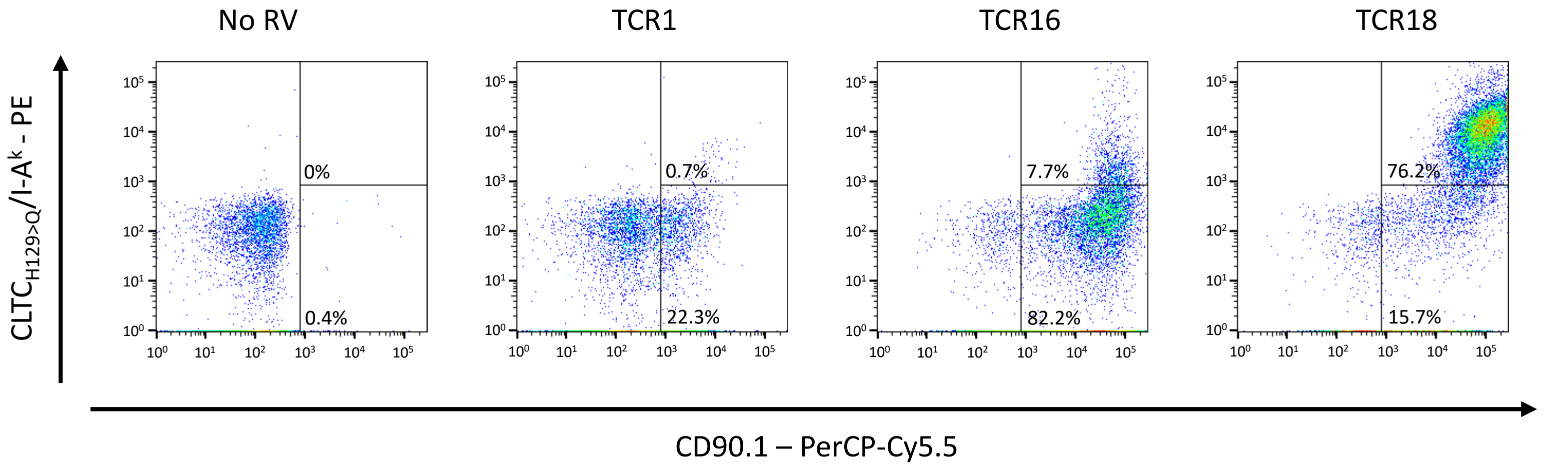

Table 1

Clone ID	Clone #	TRAV	TRAJ	TRBV	TRBJ
TCR1	2	TRAV9-4	TRAJ5	TRBV13-1	TRBJ1-1
TCR2	5	TRAV9-4	TRAJ5	TRBV13-1	TRBJ1-1
TCR3	11	TRAV13-4-DV7	TRAJ27	TRBV10	TRBJ1-4
TCR4	9	TRAV14D-1	TRAJ21	TRBV13-3	TRBJ1-2
TCR5	8	TRAV4-2	TRAJ26	TRBV1	TRBJ2-5
TCR6	6	TRAV7D-5	TRAJ15	TRBV2	TRBJ1-5
TCR7	6	TRAV7D-4	TRAJ7	TRBV5	TRBJ2-5
TCR8	5	TRAV7-1	TRAJ37	TRBV2	TRBJ2-5
TCR9	5	TRAV6D-4	TRAJ43	TRBV12-2 + TRBV13-2	TRBJ1-4
TCR10	5	TRAV3-4	TRAJ40	TRBV12-2 + TRBV13-2	TRBJ2-7
TCR11	4	TRAV7D-5	TRAJ37	TRBV2	TRBJ2-1
TCR12	4	TRAV14D-1	TRAJ21	TRBV13-3	TRBJ1-4
TCR13	4	TRAV14D-2	TRAJ9	TRBV31	TRBJ1-3
TCR14	4	TRAV4-3	TRAI9	TRBV1	TRABJ1-3
TCR15	5	TRAV14D-2	TRAJ40	TRBV1	TRBJ2-7
TCR16	1	TRAV6N-7	TRAJ57	TRBV2	TRBJ2-7
TCR17	1	TRAV7-1	TRAJ37	TRBV2	TRBJ2-7
TCR18	1	TRAV7-3	TRAJ15	TRBV2	TRBJ2-7
TCR19	7	TRAV4-2	TRAJ37	TRBV20	TRBJ1-6
TCR20	6	TRAV6-2	TRAJ6	TRBV20	TRBJ2-7

Table 2

Clone ID	Clone #	CD90.1 Expression	Tetramer Binding
TCR1	2	Yes	Yes
TCR2	5	Yes	Yes
TCR3	11	Yes	No
TCR4	9	Yes	No
TCR5	8	Yes	No
TCR6	6	Yes	Yes
TCR7	6	Yes	No
TCR8	5	Yes	Yes
TCR9	5	Yes	Yes
TCR10	5	Yes	No
TCR11	4	Yes	Yes
TCR12	4	No	No
TCR13	4	Yes	Yes
TCR14	4	Yes	Yes
TCR15	5	Yes	No
TCR16	1	Yes	Yes
TCR17	1	Yes	Yes
TCR18	1	Yes	Yes
TCR19	7	Yes	No
TCR20	6	Yes	No
